# Electroencephalographic abnormalities and clinical phenotypes in children with autism spectrum disorder: a single center cohort study

**DOI:** 10.3389/fneur.2026.1831392

**Published:** 2026-05-29

**Authors:** Natalia Wizner, Michał Wizner, Julia Rokosz, Magdalena Matlakiewicz, Magdalena Hankus, Justyna Paprocka

**Affiliations:** 1 Department of Pediatric Neurology, Faculty of Medical Sciences, Students’ Scientific Society, Medical University of Silesia, Katowice, Poland; 2 Department of Pediatric Neurology, Faculty of Medical Sciences, Medical University of Silesia, Katowice, Poland

**Keywords:** ASD, children, EEG, epilepsy, intellectual disability, sleep disorders

## Abstract

**Background:**

Electroencephalographic (EEG) abnormalities are frequently observed in children with autism spectrum disorder (ASD), even in the absence of clinical seizures. However, the clinical significance of different EEG patterns in ASD remains incompletely understood.

**Objective:**

To investigate associations between EEG abnormalities and selected clinical characteristics in children with ASD.

**Methods:**

This study analyzed medical records of 180 children with ASD hospitalized at the Pediatric Neurology Department at the Upper Silesian Child Health Center in Katowice. Patients were stratified by epilepsy diagnosis and EEG characteristics (normal, non-paroxysmal changes, paroxysmal changes). Clinical variables analyzed included developmental milestones, intellectual disability severity, sleep disturbances, hyperactivity, sensory integration disorders, aggressive behaviors, and motor deficits. Statistical analysis employed Mann–Whitney *U* test, Kruskal-Wallis test, and Fisher’s exact test as appropriate.

**Results:**

Sleep disorders showed significant association with EEG pattern type (*p* = 0.041), occurring most frequently in patients with non-paroxysmal changes (20%) compared to those with paroxysmal changes (5.9%) and normal recordings (7%). Children with comorbid epilepsy demonstrated significantly higher rates and severity of intellectual disability compared to those without epilepsy (*p* = 0.004 and *p* = 0.007, respectively). Paroxysmal abnormalities were more prevalent in the epilepsy group (62% versus 38%, *p* = 0.01). After adjusting for age, no significant associations were found between epilepsy diagnosis or EEG abnormalities and speech delay, aggression, sensory integration disorders, or motor deficits.

**Conclusion:**

Non-paroxysmal EEG abnormalities may represent a distinct neurophysiological correlate of sleep disorders in children with ASD. Comorbid epilepsy is strongly associated with intellectual disability severity, supporting the need for comprehensive neurological evaluation in this population. While broad categorical EEG patterns did not reveal significant associations with most clinical manifestations in our sample, more granular EEG analysis may detect subtle correlations not apparent with our simplified classification approach.

## 1 Introduction

Autism spectrum disorder (ASD) is a neurodevelopmental disorder characterized by deficits in social communication and the presence of restricted interests and repetitive behaviors (1). According to a systematic review by Zeidan et al. (2) approximately 1 in 100 children are diagnosed with ASD worldwide. The median male-to-female ratio was 4.2. The etiology of ASD appears to be multifactorial involving both genetic and environmental factors (3, 4). Approximately 20–25% of patients diagnosed with ASD have had a causal genetic variant identified (5, 6). The symptoms of ASD may usually be identified by the age of 2 years. The clinical presentation of ASD may include impairments in cognitive and communication skills, including restrictive, repetitive and stereotyped patterns of behavior, interests and activities as well as abnormal sensory responses, general loss of engagement with the environment. Furthermore, children may exhibit normal development and the presence of age-appropriate milestones up to the age of at least 2 years thus careful observation of a child’s development prior to the diagnostic process is crucial (7, 8).

According to Khachadourian et al. children with ASD had a substantially higher standardized prevalence of various comorbidities compared to their siblings without an ASD diagnosis. The most common comorbidities were attention deficit hyperactivity disorder (ADHD), learning disability and intellectual disability (ID). According to Dizitzer et al. approximately 85% of children with ASD may present symptoms of ADHD while the median percentage of autism cases with comorbid intellectual disability was 33% (2, 9, 10). Individuals with autism may also experience sleep disorders (SD) mostly related to rapid eye movement sleep (REM); these include decreased quantity, increased undifferentiated sleep, immature organization of eye movements into discrete bursts, decreased time in bed, total sleep time, REM sleep latency, and increased proportion of stage 1 sleep (11).

Electroencephalography (EEG) can serve as a tool for a deeper analysis of certain aspects of ASD. It is the method of choice for recording ictal epileptic activity, and in patients with autism, the incidence of epilepsies ranges from 18.2–42%. Additionally, both epileptic seizures and ASD may be the result of similar underlying disturbances in neurotransmission, such as abnormal GABAergic transmission, which affects the regulation of neuronal activity in the central nervous system (12, 13).

It is known that among individuals with ASD with no history of seizures, EEG results may show either paroxysmal abnormalities (focal or generalized) or non-paroxysmal abnormalities, like slow waves or asymmetry in the background rhythm. In the group of patients with ASD and abnormal EEGs but no seizures, paroxysmal abnormalities are observed in 23–80% of cases (14–18).

Some researchers suggest that EEG may be useful in distinguishing individuals with ASD and certain co-occurring conditions. However, the link between EEG abnormalities and the core features of ASD remains unclear. In some studies, paroxysmal abnormalities on EEG, even in the absence of clinical seizures, are believed to contribute to behavioral, language, and cognitive difficulties (19). Nonetheless, not all studies have supported this idea.

The review of literature from the past decade (2014–2024) on the connection between EEG changes and various disorders—such as the severity of autism-related traits, development, behavior, sleep, and movement issues—does not provide clear conclusions regarding how EEG changes affect the development of these disorders (20).

Given the current state of knowledge, there remains a clear research gap regarding the use of EEG in analyzing the correlation between specific changes in the EEG recordings and the neurophysiological aspects of ASD and its associated symptoms, which requires further exploration. The aim of our study was to analyze specific EEG patterns potentially linked to ASD and co-occurring conditions such as speech delay, intellectual disability, aggression, sensory integration dysfunctions, sleep disorders, hyperactivity, and motor disorders, and to assess their diagnostic utility.

## 2 Materials and methods

### 2.1 Study group and selection procedure

This retrospective study analyzed medical records of patients hospitalized in the Pediatric Neurology Department at the Upper Silesian Child Health Center in Katowice, Poland. The research group included children diagnosed with autism spectrum disorder. Initially, the medical records of 376 children diagnosed with ASD were screened for eligibility. Following a detailed review, 196 patients were excluded from the final analysis, primarily due to the absence of an EEG recording or duplicate entries. EEG recordings were obtained based on clinical indication as part of standard neurological evaluation, rather than as a uniform research protocol applied to all hospitalized children. This reflects real-world clinical practice where EEG examination was performed when clinical suspicion of seizure activity, neurological abnormalities, or comprehensive EEG characterization was deemed necessary by treating clinicians.

Data collection was based on the analysis of medical histories, including EEG, neurological examination, comorbidities, and assessment of patients’ psychomotor functioning. EEG recordings were performed using the international 10–20 electrode placement system. Recordings were obtained during wakefulness and, when possible, during sleep. EEG traces were visually reviewed by experienced pediatric neurologists. EEG findings were classified as normal, non-paroxysmal abnormalities, or paroxysmal abnormalities.

In the study group, the following variables were collected: demographic data (sex, age), developmental parameters (age at achievement of developmental milestones, occurrence of speech regression), and clinical presentation (degree of intellectual disability, sensory integration disorders, hyperactivity, sleep disturbances, stereotypies and other motor deficits, as well as the occurrence of aggressive behaviors). Diagnosis of autism spectrum disorder was established according to DSM-5 criteria by experienced child psychiatrists and neurologists.

Inclusion criteria were: (1) age 0–18 years; (2) diagnosis of ASD; and (3) at least one EEG recording performed during the hospitalization in wakefulness and/or sleep states (depending on the age and clinical picture).

### 2.2 Statistical analysis

Statistical calculations were performed in the Python programming environment (version 3.12.8) using the libraries *pandas*, *numpy*, *scipy*, *plotly*, and *matplotlib*. A statistical significance level of *p* < 0.05 was adopted.

#### 2.2.1 Descriptive statistics

Categorical (qualitative) variables were described using counts (n) and percentages (%). Continuous (quantitative) variables were presented using the median (Me) and interquartile range (Q1–Q3), which was dictated by the skewness of the distributions (verified by the Shapiro–Wilk test and Levene’s test for homogeneity of variance).

#### 2.2.2 Statistical inference

- Comparison of groups for continuous variables: The choice between parametric tests (Student’s *t*-test and ANOVA) and non-parametric tests (Mann–Whitney *U* test and Kruskal-Wallis test) was made based on verification of assumptions about normality of distributions and homogeneity of variance.
- Analysis of categorical variables: Relationships were examined using contingency table analysis, applying Pearson’s Chi-square test, Chi^2^ test with Yates’ correction, or Fisher’s exact test, depending on the expected cell frequencies.

In situations where the data structure made it impossible to meet the rigorous test assumptions (e.g., in multi-field analyses with low frequencies), the analysis was conducted in override mode, and the obtained results were treated as indicative, recommending caution in their interpretation.

In instances where the data structure prevented meeting rigorous test assumptions (e.g., in multi-field analyses with low frequencies), the analysis was conducted in ‘override mode’. These specific cases are explicitly identified and described later in the Results section; accordingly, the findings were treated as indicative, and caution is recommended in their interpretation.

## 3 Results

Characteristics of the study group are presented in Table 1.

**Table 1:** Characteristics of the study population.

Characteristic	Value/count
Study group size (*N*)	180
Gender (boys)	133 (73.9%)
Age [years] (mean, range)	7.5 (1.5–17.5)
Epilepsy diagnosis	60 (33.3%)
Birth weight [g] (mean, range)	3,257 (890–5,300)
Paroxysmal EEG abnormalities	61 (33.9%)
Non-paroxysmal EEG abnormalities	65 (36.1%)

**Figure 1:**
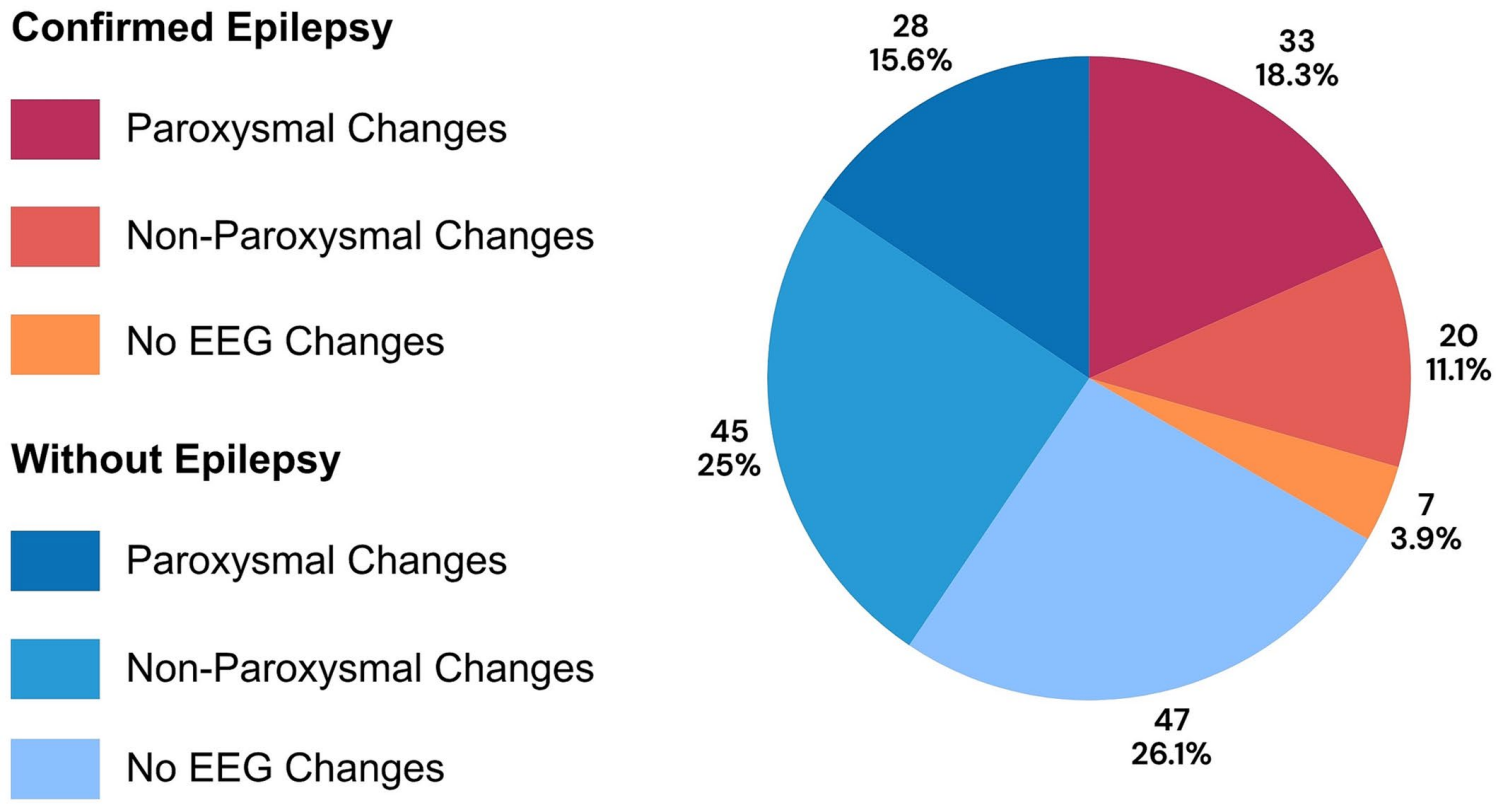
Study population distribution pie chart.

**Figure 2:**
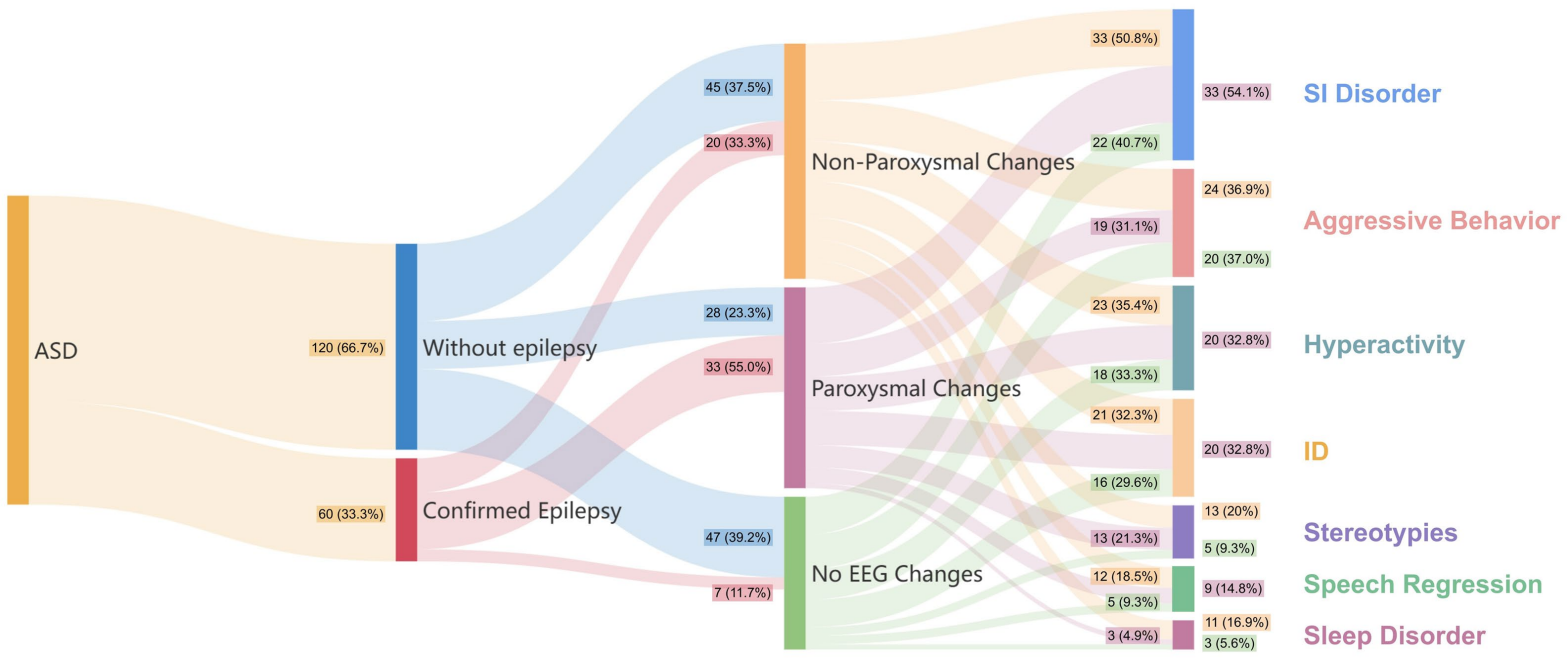
Sankey diagram illustrating the relationship between epilepsy diagnosis, EEG patterns, and clinical phenotypes in children with ASD. ASD, autism spectrum disorder; ID, intellectual disability; SI, sensory integration. Numerical values represent the absolute number of subjects ( n ); percentages in parentheses indicate the proportion relative to the specific source subgroup (node) from which the flow originates.

For the purpose of conducting a detailed comparative analysis, patients were divided into subgroups based on two criteria: clinical diagnosis of epilepsy and EEG recording characteristics. The scheme of this division is presented in Figure 1. Figure 2 presents a Sankey diagram summarizing the clinical distribution of the study cohort. It maps the flow from epilepsy diagnosis and EEG patterns to various comorbidities, with band widths proportional to patient frequency in each subgroup. This visualization highlights the complex overlap between neurophysiological findings and associated clinical phenotypes.

Table 2 provides a detailed characterization of the EEG findings, illustrating the distribution of focal, generalized, and combined patterns among patients with paroxysmal and non-paroxysmal abnormalities.

**Table 2:** Distribution of focal, generalized, and combined EEG patterns.

EEG finding	Subtype	*N*
Paroxysmal		61 (100%)
	Focal	17 (27.9%)
	Generalized	32 (52.5%)
	Combined generalized and focal	11 (18%)
Non paroxysmal		65 (100%)
	Focal	17 (26.2%)
	Generalized	35 (53.9%)
	Combined generalized and focal	11 (16.9%)

### 3.1 Comparison of the study cohort based on EEG findings

**Figure 3:**
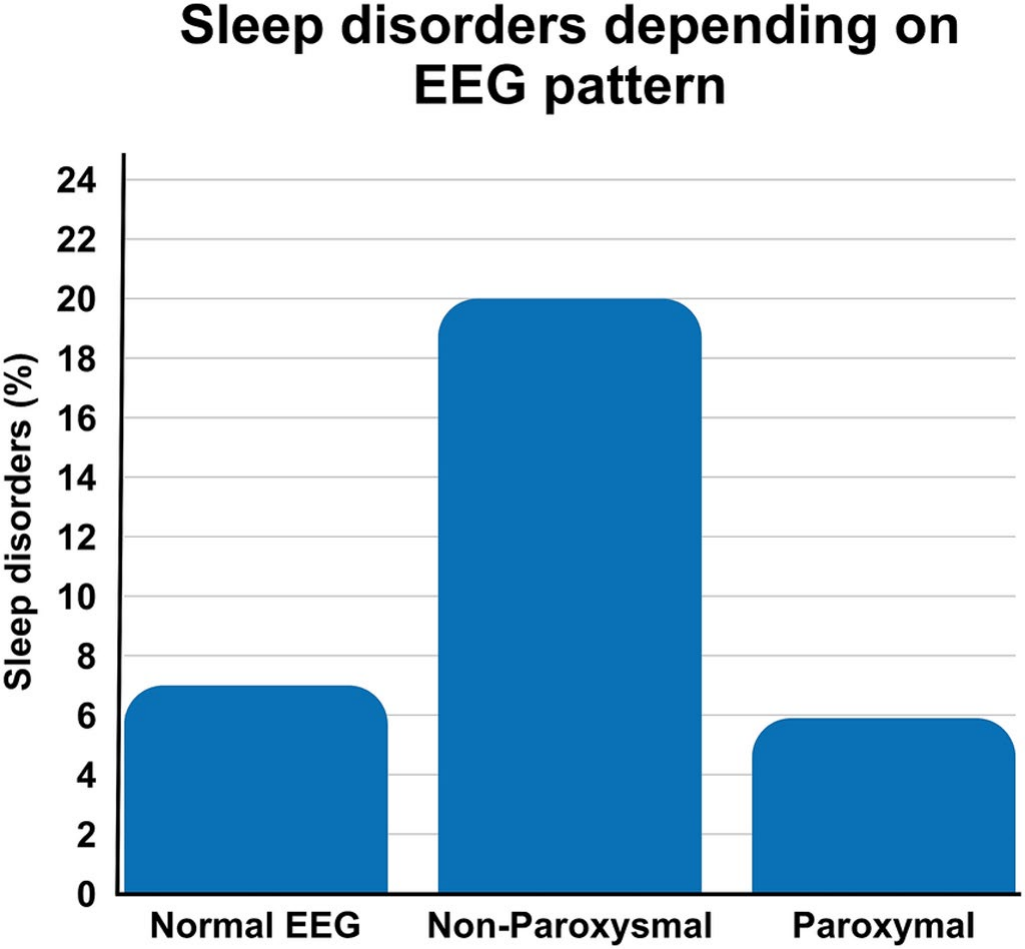
Sleep disorders depending on EEG pattern.

Analysis of the relationship between the type of EEG recording (normal, non-paroxysmal changes, paroxysmal changes) and selected clinical variables in the entire study population showed a statistically significant difference only in the area of sleep disorders (*p* = 0.041), which is presented in Figure 3.

**Figure 4:**
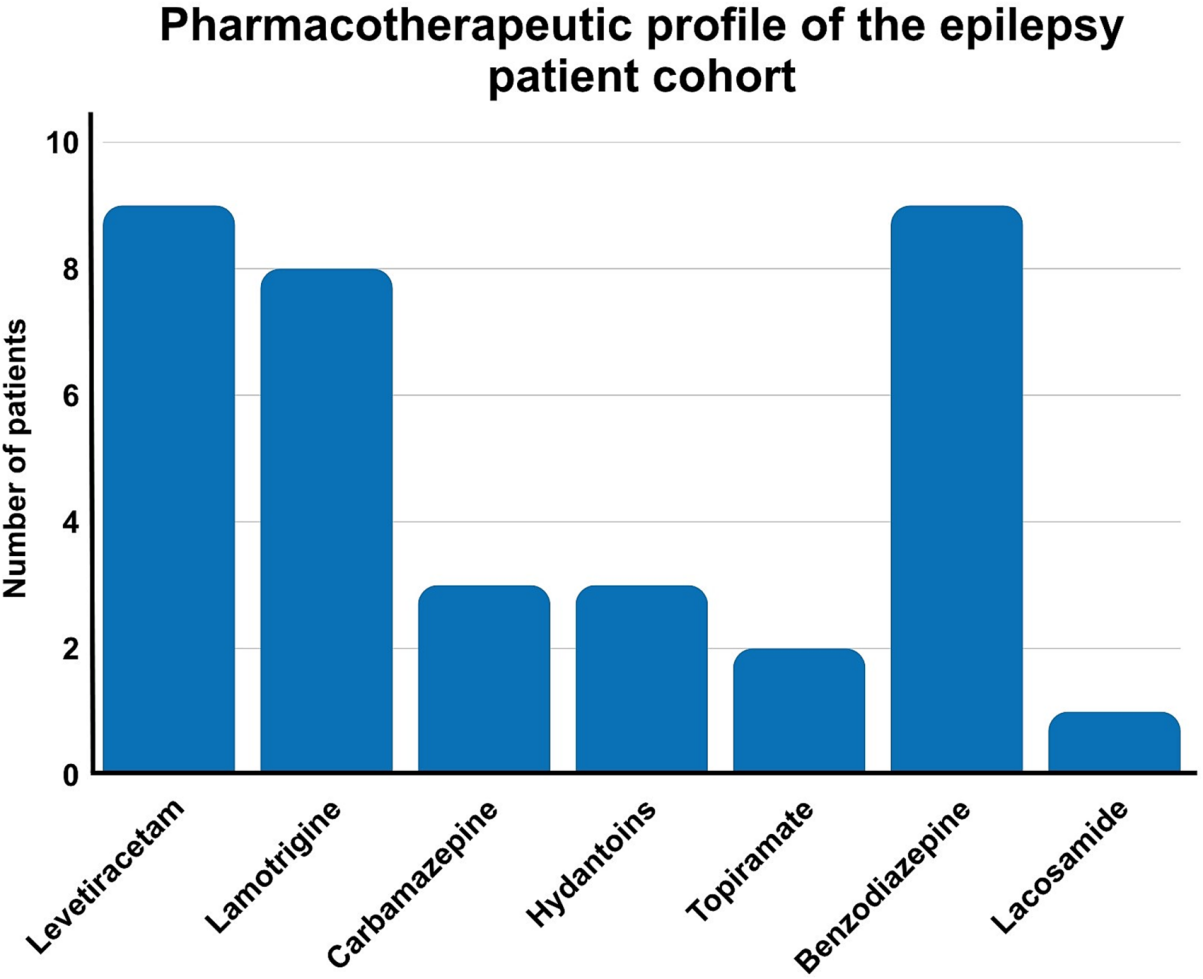
Distribution of antiepileptic drugs (AEDs) used by patients with epilepsy.

Detailed post-hoc analysis (Fisher’s exact test) revealed that SD occurred most frequently in the group of patients with non-paroxysmal changes (20%). This frequency was significantly higher compared to the group with normal recordings (7%, *p* = 0.049) and the group with paroxysmal changes (5.9%, *p* = 0.029). However, no significant differences were noted in the occurrence of sleep disorders between patients with normal EEG and patients with paroxysmal changes. To exclude the potential confounding influence of age and pharmacotherapy, a multivariate logistic regression model was employed. The analysis, which accounted for age and the use of antiepileptic, psychiatric, and sedative medications, confirmed that non-paroxysmal EEG abnormalities remain independently associated with the occurrence of sleep disorders (*p* = 0.04; OR = 0.08). Age did not reach statistical significance (*p* = 0.14), and none of the medication classes- antiepileptic, psychiatric, or sedative drugs- were found to be independent predictors of sleep disturbances (all *p* > 0.97). Figure 4 presents the distribution of antiepileptic drugs used by the patients.

Notably, the chi-square override mode was specifically applied to the analyses of sleep disorders, intellectual disability, motor disorders, and speech delay (first words), as these subgroups did not meet all rigorous test assumptions; consequently, these results should be treated as indicative and exploratory. For the remaining clinical variables-including aggression, sensory integration (SI) disorders, speech regression, and hyperactivity-standard test assumptions were satisfied, and no statistically significant associations with EEG patterns were found (all *p* > 0.05).

### 3.2 Clinical comparison of groups stratified by epilepsy diagnosis

In this analysis, patients without recorded abnormalities in EEG (*n* = 54) from both groups were excluded. This exclusion is justified by several methodological considerations: Normal EEG results constituted significantly different proportions in each group (11.7% vs. 39.2%), which could introduce bias; small sample size in the epilepsy/normal EEG subgroup (*n* = 7) limited statistical reliability; maintaining symmetry of comparison by including only patients with documented EEG changes allowed a more precise analysis of pathological patterns.

**Figure 5:**
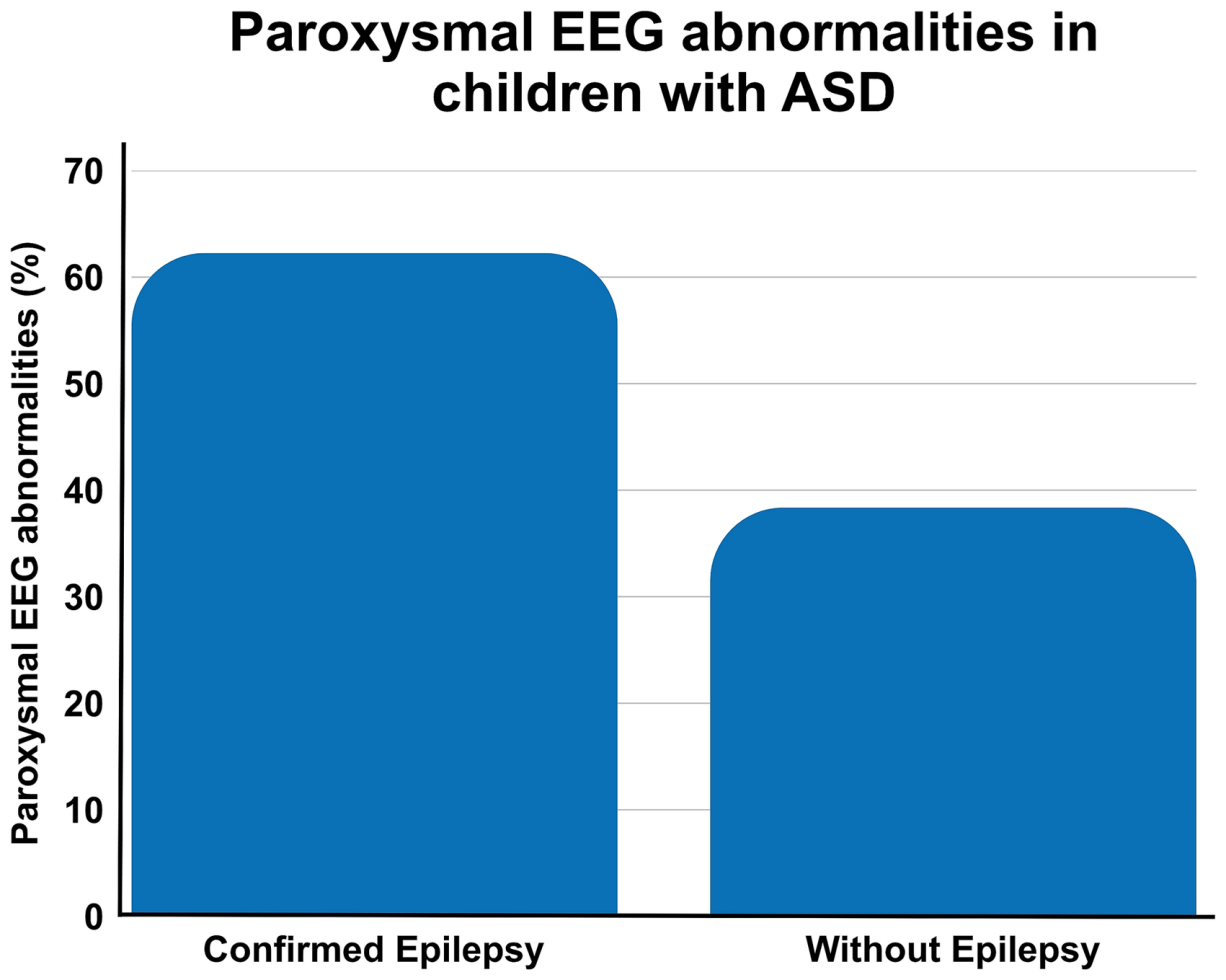
Paroxysmal EEG abnormalities in children with ASD with and without epilepsy.

Statistically significant differences between these two groups were observed for the occurrence of intellectual disability and its severity. Although initial analysis suggested a difference in the absence of speech (*p* = 0.03), this association did not remain statistically significant after adjusting for age differences between the groups (*p* > 0.05). Detailed results are presented in Table 3. The statistically significant difference regarding the occurrence of paroxysmal changes is shown in Figure 5.

**Table 3:** Comparison of patient characteristics with epilepsy vs. without epilepsy.

Characteristic		Without epilepsy *N* = 73	Epilepsy *N* = 53	*p*-value
Intellectual disability	Mild	11 (21%)	12 (30%)	0.007
	Moderate	4 (7.5%)	8 (20%)	
	Severe	1 (1.9%)	5 (13%)	
Speech delay (absence of spoken words)		15 (23%)	3 (6.7%)	0.03*

*Not significant after age adjustment.

No statistically significant differences were found between the groups in terms of sex, age of starting to walk, occurrence of aggressive behaviors, sensory integration disorders, hyperactivity, stereotypies, or sleep disorders.

### 3.3 Subgroup analysis of EEG patterns (paroxysmal vs. non-paroxysmal) within cohorts with and without comorbid epilepsy

In order to verify whether the type of EEG changes (paroxysmal vs. non-paroxysmal) itself differentiates patients within the same diagnosis, within-group analyses were performed:

- In the group of patients without epilepsy: Comparison of children with non-paroxysmal and paroxysmal changes showed no significant differences in any of the examined clinical parameters. Only in the case of age of starting to walk (*p* = 0.06) and SD (*p* = 0.08) were trends close to significance observed, however not exceeding the adopted threshold of *p* = 0.05.
- In the group of patients with epilepsy: Similarly, division into paroxysmal and non-paroxysmal changes did not differentiate the group in terms of severity of developmental, cognitive, or behavioral disorders.

### 3.4 Characteristics of patients with paroxysmal EEG abnormalities, stratified by comorbid epilepsy status

The final element of the analysis was the comparison of children with diagnosed epilepsy and children without a diagnosis of epilepsy, in whom, however, paroxysmal changes were recorded in the EEG. The only statistically significant difference concerned intellectual disability, which occurred more frequently in the group with epilepsy (*N* = 14, 56%) compared to without epilepsy (*N* = 6, 26%), *p* = 0.036. The remaining data are detailed in Table 4.

**Table 4:** Clinical characteristics of patients with paroxysmal abnormalities, stratified by the presence of comorbid epilepsy.

Characteristic		Without epilepsy *N* = 28	Epilepsy *N* = 33	Test statistic	*p*-value
Sex (M)		22 (79%)	24 (73%)	$\chi^{2}\;$ = 0.05	0.82
Age [mo.]		61 (38, 89)	99 (54, 150)	U = 275	0.007
Walking [mo.]		15.5 (13.0, 18.0)	16.0 (13.5, 18.0)	U = 355.5	0.89
Speech-words [mo.]		21 (12, 24)	15 (12, 36)	U = 252	>0.9
Speech-sentences [mo.]		48 (36, 60)	34 (24, 60)	U = 85	0.47
Speech regression		3 (15%)	6 (25%)	—	0.66
Intellectual disability	Mild	3 (13%)	8 (32%)	$\chi^{2}\;$ = 4.68	0.2
	Moderate	2 (8.7%)	3 (12%)		
	Severe	1 (4.3%)	3 (12%)		
Aggressive behavior		12 (43%)	7 (21%)	$\chi^{2}$ = 2.38	0.12
Sensory integration disorders		18 (64%)	15 (45%)	$\chi^{2}$ = 1.47	0.23
Hyperactivity		9 (32%)	11 (33%)	$\chi^{2}\;$ = 0	>0.9
Sleep disturbances		1 (3.6%)	2 (6.3%)	$\chi^{2}\;$ = 0	>0.9
Stereotypies		7 (25%)	6 (18%)	$\chi^{2}$ = 0.11	0.74
Other motor disorders		5 (18%)	9 (27%)	$\chi^{2}$ = 0.32	0.57
Motor delay		1 (3.7%)	2 (6.7%)	—	>0.9
Speech delay (absence of spoken words)		5 (20%)	3 (11%)	—	0.45
Speech delay (absence of simple sentences)		11 (55%)	7 (30%)	$\chi^{2}$ = 1.74	0.19

U , Mann–Whitney U test statistic; $\chi^{2}$ , Chi-square test statistic.

## 4 Discussion

Over the past two decades, there has been a steady increase in the prevalence of ASD, with the most recent estimates indicating that it affects approximately 1 in 36 children, although establishing the diagnosis often remains a clinical challenge (21, 22). The diagnosis of ASD is associated with a higher risk of other chronic diseases, particularly neurological ones, including epilepsy (23). According to a meta-analysis from 2021, the prevalence of epilepsy in this population is 7% (95% CI: 4–11), compared to the general population of approximately 1%, and EEG abnormalities themselves are even more common (24–26). In turn, the percentage of ASD diagnoses in patients with epilepsy ranges from 15% to as much as 74% (27). Currently, the diagnosis of ASD is based on behavioral methods, such as the Autism Diagnostic Observation Schedule (ADOS), Modified Checklist for Autism in Toddlers (M-CHAT), and Autism Diagnostic Interview - Revised (ADI-R) (28, 29). The growing application of neuroimaging, including EEG, provides objective data on the functional activity of the brain (30, 31).

Despite the strong association between epilepsy and ASD, there is a limited number of studies dedicated to the potential relationships between EEG abnormalities in children and the clinical presentation of ASD. Some studies suggest that abnormalities in the EEG recording may be helpful in identifying subgroups of patients with ASD, and some reports indicate that epileptiform abnormalities, even of a subclinical nature, may constitute a neurophysiological correlate for behavioral, linguistic, and cognitive dysfunctions (17, 19). Importantly, our study addresses this gap by analyzing a relatively large single-center cohort of 180 children with ASD, which is larger than many previous reports, where sample sizes often did not exceed 75 participants (17, 32). This sample size enhances the robustness of our estimates and allows a more reliable exploration of potential associations between clinical features and EEG patterns, although further confirmation in independent cohorts remains necessary. Existing studies involving larger cohorts of children with ASD have consistently documented a high prevalence of EEG abnormalities, yet many do not systematically examine how specific electrophysiological findings relate to distinct ASD-related symptoms or comorbidities, as we attempted in the present analysis (16, 33). This lack of detailed correlation data contributes to clinical uncertainty, particularly when deciding whether subclinical epileptiform discharges in children without overt seizures should prompt therapeutic intervention.

Sleep disorders are among the most frequently co-occurring problems in children with neurodevelopmental disorders (NDD), reported in over 86% of them (34). In the pediatric population with ASD, this frequency reaches up to 80%, and in children with ASD and comorbid epilepsy, it increases to 50–95% (35–39). These disorders, manifesting as difficulties with sleep onset, sleep maintenance, nocturnal awakenings, and sleep apnea, are bidirectional in nature: poor sleep exacerbates core ASD symptoms, such as restricted and stereotyped behaviors and social and communication deficits, while the underlying conditions contribute to sleep disturbances (38, 40–45).

Sleep problems in this population are complex and often result from neurobiological and genetic alterations, such as abnormal expression of neurotransmitters (serotonin, melatonin, GABA) involved in sleep regulation, or dysregulation of the autonomic nervous system caused by sensory hyperreactivity (43, 46–50). Moreover, children with ASD exhibit differences in central nervous system organization that may alter sleep macrostructure (e.g., shortened REM sleep) and melatonin secretion abnormalities (51). Among patients with epilepsy, children with comorbid ASD are particularly vulnerable to insomnia (52, 53). Although nocturnal seizures may disrupt sleep, it has been suggested that behavioral problems may reflect coexisting sleep disorders rather than seizure frequency alone, which is consistent with the observation that sleep disturbances occur even in patients with epilepsy without nocturnal seizures (53–55). Treatment with antiepileptic drugs (AEDs) can also affect sleep (e.g., GABAergic AEDs may shorten sleep latency and induce daytime somnolence, while others may cause insomnia) (53, 56).

In light of these data, the results of the present study are particularly interesting. In our cohort, EEG pattern type was associated only with sleep disorders (*p* = 0.041), which occurred most frequently in the subgroup of patients with non-paroxysmal abnormalities (20%), compared to the group with paroxysmal changes (5.9%) and no abnormalities (7%). Notably, such a correlation was not demonstrated for the diagnosis of epilepsy itself. This finding, combined with the scarcity of reports regarding the association of SD directly with non-paroxysmal abnormalities, provides new information about this relationship. Sleep disorders represent a frequent comorbidity with ASD, and atypical delta wave activity during NREM sleep, suggesting abnormal cortico-thalamic connectivity, has been previously described in patients with ASD (20, 57).

Although the prevalence of sleep disturbances in our study (5.9–20%) was substantially lower than reported in prospective and cross-sectional studies (50–95%) evaluating children with epilepsy, this likely reflects our reliance on retrospective hospital records and the absence of standardized, dedicated sleep assessment tools (35–39, 58). This methodology primarily captures clinically significant, severe cases that were explicitly reported during consultations, potentially omitting milder presentations that did not prompt clinical documentation. Importantly, despite this underestimation, the differential prevalence between EEG pattern groups remains clinically meaningful, as it identifies children with ASD and non-paroxysmal changes as more likely to have documented sleep disturbances.

A comparison of patients with and without epilepsy indicated that the presence of epilepsy was positively associated with the severity and frequency of intellectual disability. Individuals with ASD and comorbid epilepsy tend to exhibit higher rates of ID and broader neurodevelopmental impairments—including deficits in speech, adaptive functioning, and psychiatric status—compared to those with ASD without epilepsy, underscoring the importance of timely seizure detection and treatment (18, 27, 59). Cross-sectional analyses, including 5,815 children with ASD, have shown that both age and cognitive abilities are independently linked to epilepsy (60). In a cohort of 6,975 children with ASD, ID emerged as the strongest predictor of epilepsy risk, independent of age and sex, and further functioned as an independent risk marker for both ASD and epilepsy (61). Other analyses support this association, suggesting that higher intelligence quotient was associated with a lower probability of developing epilepsy. For example, a meta-analysis of children with ASD under 12 years of age showed that the prevalence of epilepsy was 21% in those with comorbid ID, compared to 8% in children without ID, with the highest rates observed in children with more severe ID (IQ < 40) (62). It is worth adding that both the diagnosis of ASD itself and EEG abnormalities in general may negatively affect intelligence quotient (63, 64). Developmental delay and intellectual disability, often referred to as developmental encephalopathies, may manifest as early as the first months of life. Despite the diversity of symptoms, 22.2% of children with intellectual disability also suffer from some form of epilepsy (65). The term epileptic encephalopathy (EE) was introduced to describe conditions in which epileptic activity itself is considered the cause of developmental impairment. Epileptic encephalopathies are often based on genetic variants, where increased predisposition to seizures is associated with deterioration of cognitive functions.

Although a child’s development is independent of epileptic seizures, their activity may exacerbate pre-existing developmental delays. In this context, simultaneous worsening of interictal EEG abnormalities is not always recorded, and pathophysiological evidence for their association with developmental deterioration is limited (66).

Initial analysis indicated that the delayed onset of first words was more prevalent in the no-epilepsy group compared to the epilepsy group (23% vs. 6.7%). However, after adjusting for the substantial age difference between the cohorts (mean age 55 vs. 99 months), this association was no longer statistically significant. This suggests that the initial finding was primarily confounded by the higher mean age in the epilepsy group, which likely provided these children with more time for early delays to resolve or for children to “catch up” in their speech development (67). Consequently, epilepsy status does not appear to be an independent predictor of early speech delay in this population when age is taken into account.

In children admitted for suspected epilepsy, a major diagnostic challenge is that abnormal EEG patterns may also be observed in otherwise healthy individuals, and when combined with the frequent over-interpretation of paroxysmal events by caregivers, this necessitates careful clinical judgment to determine which findings are truly clinically relevant and warrant medical intervention (68). In children with ASD, EEG abnormalities in the absence of clinical seizures are even more common, which was also reflected in our cohort, where paroxysmal changes were present in 23.3% of all patients without epilepsy and in 55% of those with comorbid epilepsy (33, 69).

To maintain adequate statistical power and ensure the reliability of the findings, the primary analysis in this study was restricted to comparisons based on epilepsy diagnosis and broad EEG patterns (paroxysmal versus non-paroxysmal changes). Further subdivision of the cohort based on specific localization, discharge frequency, or a focal-versus-generalized distinction would have resulted in very small subgroups, particularly within a single-center cohort of 180 patients. It is possible that this simplified classification contributed to the predominantly negative results observed for most clinical parameters. While broader categories are necessary for statistical robustness in this sample size, they may mask subtle, phenotype-specific associations that only become apparent with more granular neurophysiological data.

Interestingly, comparison of subgroups with paroxysmal and non-paroxysmal abnormalities did not reveal any statistically significant differences, either in the group with epilepsy or without it. These findings indicate that at the current level of categorical resolution, broad EEG patterns do not show a statistically significant correlation with the frequency of typical ASD symptoms. This result provides a valuable baseline, suggesting that any existing neurophysiological correlates are likely more subtle or domain-specific, requiring higher-resolution analysis to be detected.

### 4.1 Limitations and directions for further research

This study has several important limitations. First, the retrospective design precludes establishment of direct causal relationships. This retrospective nature also meant that standardized measures of ASD severity and comprehensive etiological data were unavailable for the entire cohort, precluding their inclusion in the confounding analysis.

Second, EEG recordings were performed based on clinical indication rather than systematic research protocol. Consequently, not all hospitalized children underwent EEG evaluation, and the availability of EEG recordings likely reflects clinical suspicion of seizure activity or neurological concerns rather than standardized assessment. This introduces selection bias, as children with more obvious neurological symptoms were presumably more likely to undergo EEG evaluation. Similarly, the lack of standardized psychometric tools for assessing comorbidities such as sleep disorders is a significant limitation; relying solely on retrospective clinical documentation may lead to underreporting and underestimation of sleep disturbance prevalence in this population.

Third, the analysis of associations between sleep disorders and EEG changes revealed violations of statistical assumptions underlying the chi^2^ test, necessitating cautious interpretation of significance levels. Additionally, our simplified EEG classification (normal, non-paroxysmal, paroxysmal)- while necessary to maintain statistical power- represents a lack of a highly sensitive EEG scoring system, which may have masked more granular associations related to specific discharge localization or frequency, potentially contributing to predominantly negative findings. This foundational approach identifies a clear need for future research to implement more highly resolved analyses by stratifying cognitive functions into finer categories and adopting standardized, comprehensive scoring criteria for EEG data, such as the SCORE (Standardized Computer-based Organized Reporting of EEG) system (70). Adopting such standardized and sensitive criteria would enable a more precise analysis of potential correlations between specific neurophysiological features and cognitive or behavioral phenotypes in ASD, which our current broad-category analysis might have overlooked.

Future research should employ prospective designs with larger, more homogeneous samples and incorporate advanced quantitative EEG analysis techniques (spectral analysis, coherence analysis, event-related potentials) to enable more precise characterization of relationships between specific EEG abnormalities and clinical characteristics of children with ASD.

## Data Availability

The raw data supporting the conclusions of this article will be made available by the authors, without undue reservation.
